# Supplementary material for: Non-optical, label-free electrical capacitance imaging of microorganisms
Source: mBio. 2025 Aug 18;16(9):e01676-25. doi: 10.1128/mbio.01676-25 (PMC12421826; doi:10.1128/mbio.01676-25)
Supplement: Supplemental Material — Supplementary figures, tables, and text. [file mbio.01676-25-s0002.pdf]

SUPPLEMENTARY MATERIAL

Supplementary Table S1. Species and Strain Information

Figures	Strain	Description
Fig 1, 3<br>Fig S3, S2, S5	JMJ1061	*B. subtilis* NCIB3610 pBS32(*comI* Q12L) *lacA*::Ppen-mApple-kan<br>*amyE*::PcapB-YFP-spec Δ*sinR*::mls
Fig 2(i)	PS216	*B. subtilis* PS216<br>An undomesticated Slovenian soil isolate [1].
Fig 2(ii)	ATCC14580	*B. licheniformis* ATCC14580<br>Species type strain [2].
Fig 4	ATCC14048	*V. natriegens* ATCC14048<br>Salt marsh marine isolate, species type strain.
Fig 2(iii)<br>Fig 5<br>Fig S6	YHK038	*S. cerevisiae* BY4742 (MATα his3Δ1, leu2Δ0, met15Δ0, ura3Δ0),<br>with FLO and mating genes knocked out<br>(ΔFLO1, ΔFLO5, ΔFLO9, ΔFLO10, ΔFLO11, ΔSAG1, ΔAGA1, ΔAGA2, ΔFIG2).<br>FLO11 knocked into the URA3 locus for constitutive mNeonGreen expression<br>from the pTEF1 promoter. pTEF1-FLO11-T2a-mNeonGreen-tADH1-
Fig 5<br>Fig S6	JMJ1222	*B. subtilis* PS216 *lacA*::Pveg-mScarlet-kan.
Fig 6<br>Fig S7, S8	NCIB3610	*B. subtilis* NCIB3610<br>Species type strain [3].
Fig S4	ATCC13880	*S. marcescens* ATCC13880<br>Species type strain.

Supplementary Table S2. Sample information relevant to ECI/FL imaging

Figures	Media	Dye Name	Excitation/ Emission
Fig 1, 3, S3, S5	1% Agarose MSgg	mApple	555nm /(560nm - 839nm)
Fig 2(i)	1% Agarose MSgg	Thioflavin T	405nm /(420nm - 714nm)
Fig 2(ii), S4	1% Agarose LB	Thioflavin T	405nm /(420nm - 714nm)
Fig 2(iii)	1% Agarose YPD	mNeonGreen	492nm /(497nm - 748nm)
Fig 4	1% Agarose MLB	N/A	405nm /(420nm - 714nm)
Fig 5, S6	1% Agarose YPD	mNeonGreen	504nm /(509nm - 574nm)
Fig 5, S6	1% Agarose YPD	mCherry	587nm /(592nm - 750nm)

Supplementary Table S3. Comparison of ECI and other methods. Sizes are determined from core imaging systems only, not including installation or shared peripheral equipment such as a measurement PC. Imaging times correspond to the time to capture an area equivalent to the CMOS field of view (2.56mm × 5.12mm) at the stated resolution.

**Method**	**System Size**	**Resolution**	**Imaging Time**	**Labeling Methods Used**
ECI<br>(This Method)	0.02 m^3	10 μm	20 s	None
Macroscope<br>(Olympus MVX10)	0.4 m^3	3.2 μm<br>(2.02x/0.15)	4 ms	None,<br>Fluorescent Marker
Epifluorescence Microscope<br>(Olympus IX83)	1 m^3	1.6 μm<br>(4x/0.13)	1 s	Fluorescent Marker
Confocal Microscope<br>(Leica Stellaris 5)	2 m^3	0.71 μm<br>(25x/0.95)	40 s	Fluorescent Marker
Electron Microscope<br>(FEI Teneo SEM)	3 m^3	0.44 μm [4]	220 s [4]	None,<br>Heavy metal staining/coating

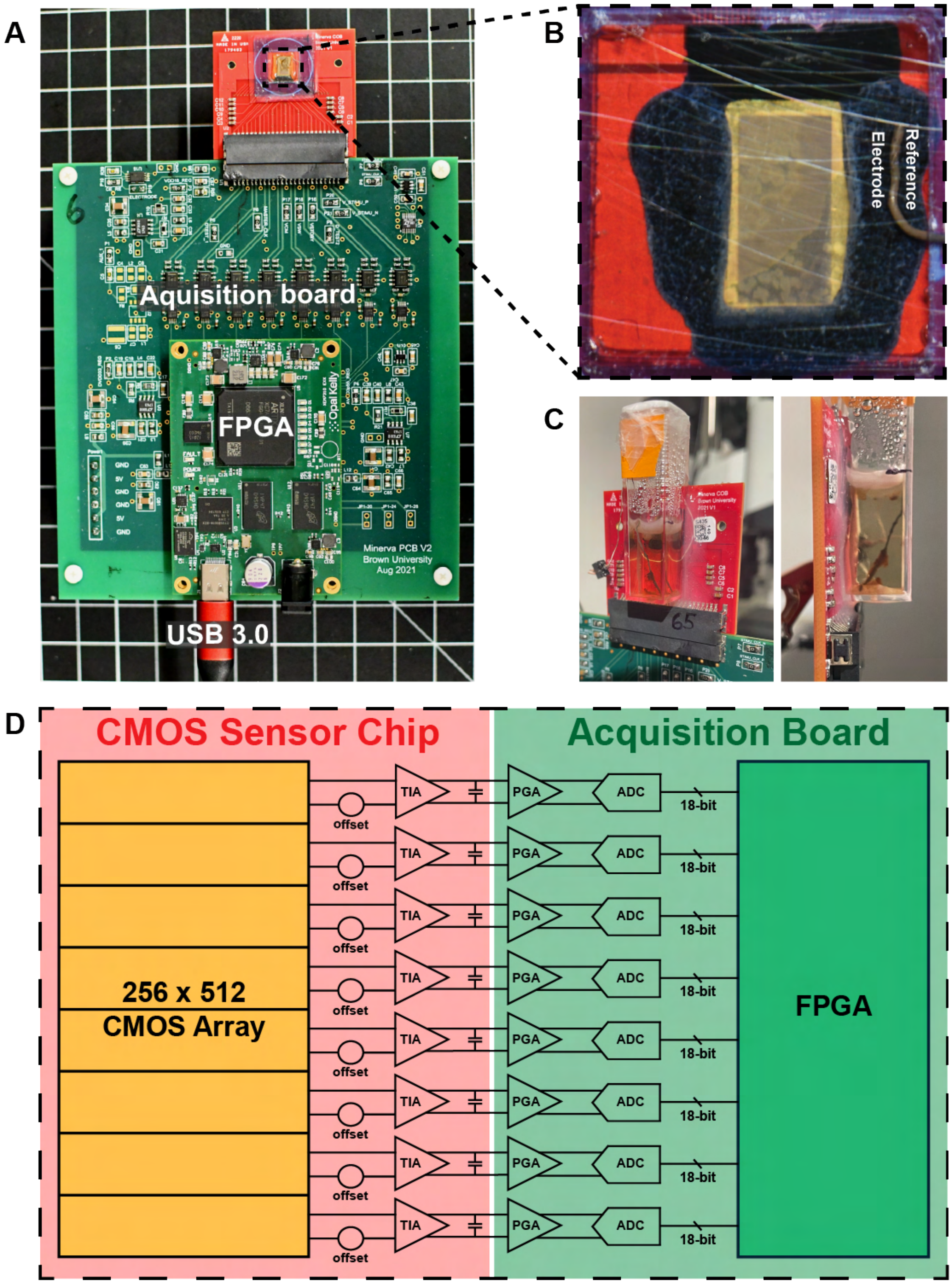

Supplementary Figure S1. **ECI acquisition hardware, and sample preparation:** (A) Data from the 131072 CMOS pixels is multiplexed into 8 readout channels, and streamed to a dedicated FPGA via a data-acquisition board. There it is decoded and transmitted via USB connection to a measurement PC, and saved in HDF5 file format. (B) The agarose sample of Fig 1D/E is seen mounted over the CMOS array, with an optional AgCl reference electrode inserted. (C) Views of the cuvette setup used in pellicle culturing, featuring the pellicle of Fig. S8 after several weeks of growth. (D) High level block diagram of system electronics, featuring the 8 readout channels. Multiplexed data from each channel undergoes Trans-impedance Amplification (TIA) followed by Programmable Gain Amplification (PGA), before signal is digitized with an 18-bit ADC on the data acquisition board, and finally decoded by the FPGA.

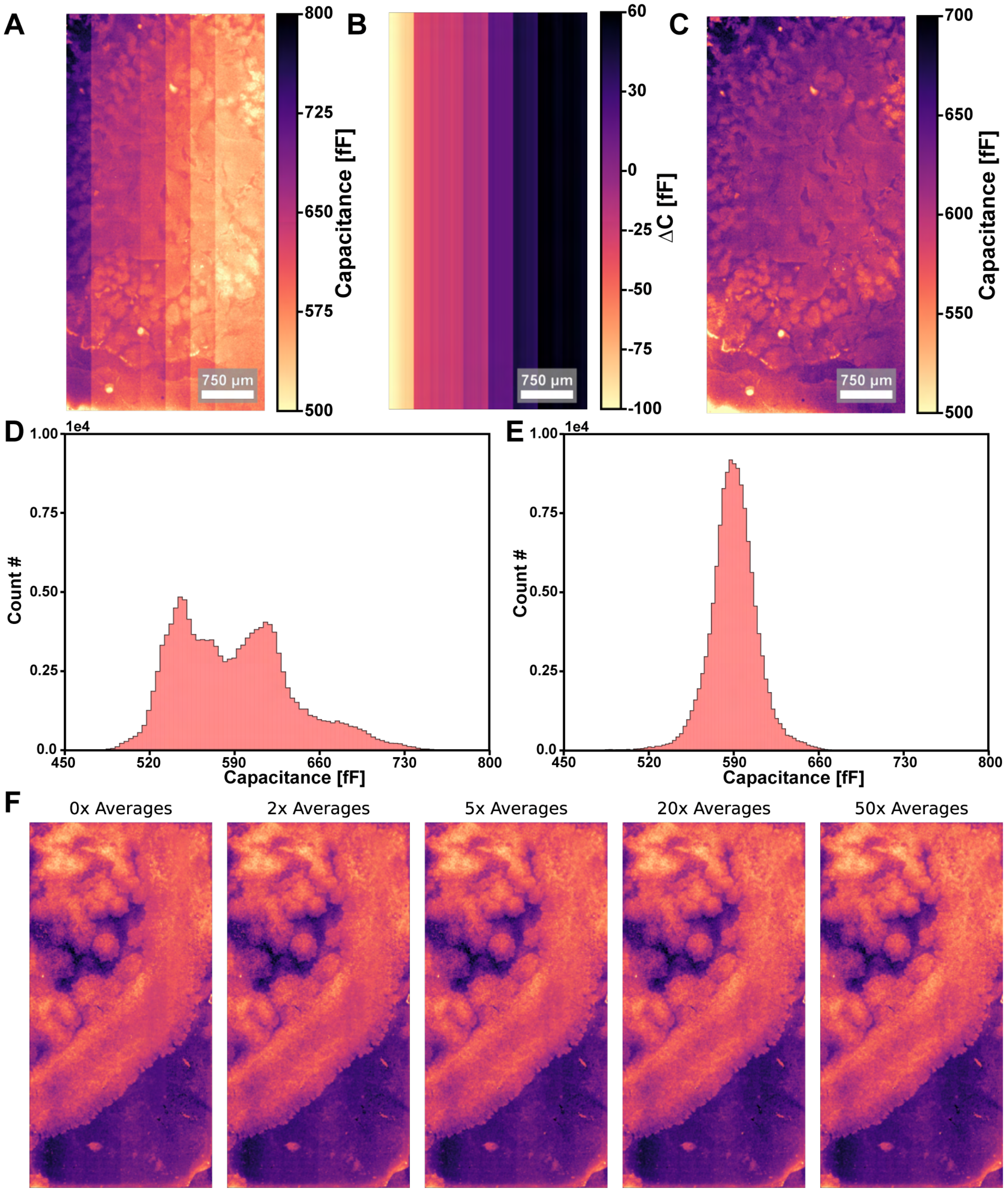

Supplementary Figure S2. Adjusting for channel readout artefacts: (A) A capacitance image with an offset artefact from the 8 channel CMOS readout. (B) The capacitance correction to be applied to the image and (C) the corrected image. (D) Histogram of capacitance values before (E) and after the correction. (F) Demonstration of the effect of frame averaging on ECI (Fig. 1 data). Image quality is improved with averaging, but not substantially beyond 5x.

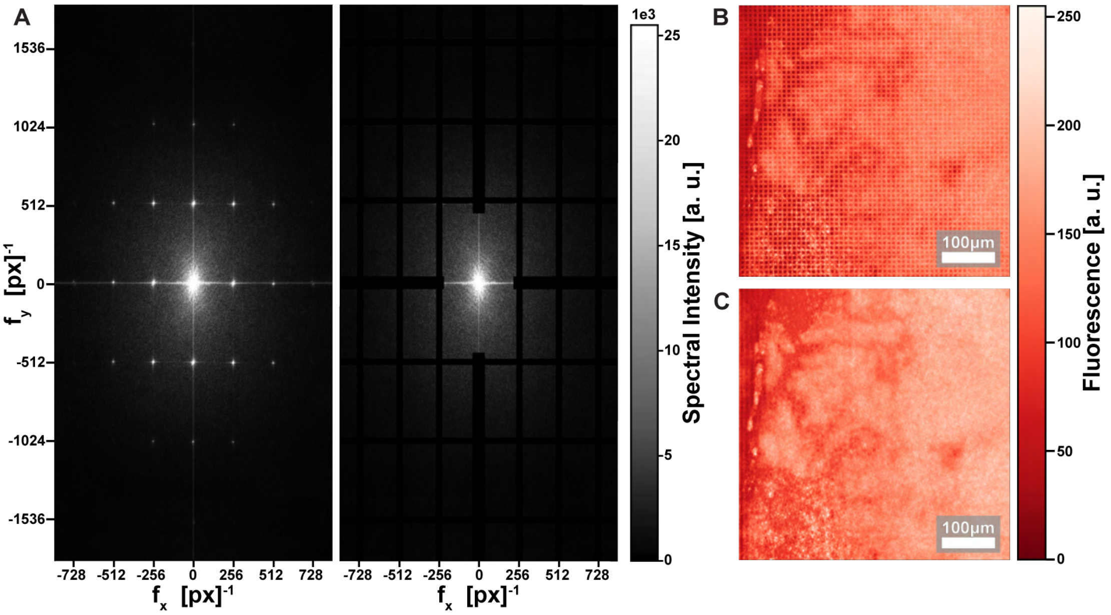

Supplementary Figure S3. FFT suppression of CMOS features in optical images: (A) The 2D FFT of a confocal image (left) with periodic features showing up at increments determined by the shape of the CMOS array (256,512). (B) A simple grid filter is applied in the frequency domain to suppress CMOS features.(C) The confocal image before (D) and after applying the FFT filter.

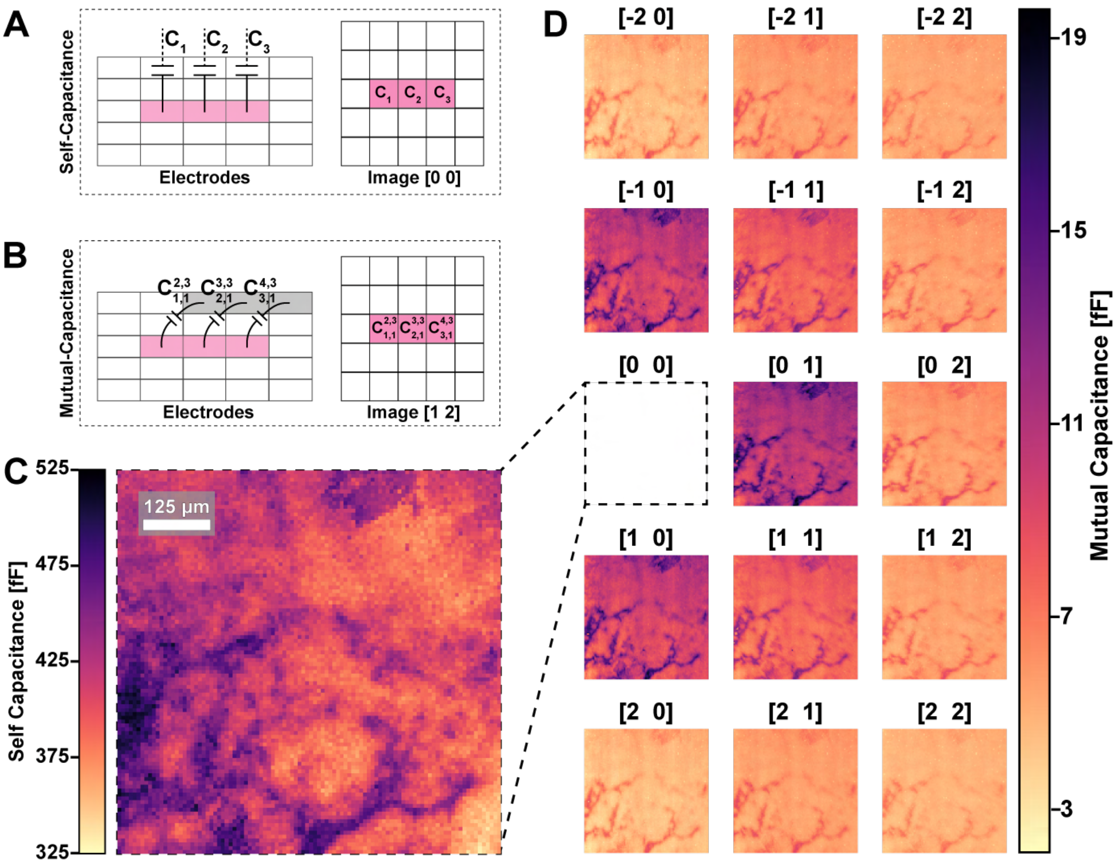

Supplementary Figure S4. Self vs mutual capacitance modalities: Our device is capable of multiple AC-coupled imaging modalities. (A) In a self-capacitance measurement, capacitance is measured between a target electrode and the rest of the array surface, while mutual capacitance measurements (B) can be made between specific electrode pairs in the array. We demonstrate these modalities with a self-capacitance image (C) of a *Serratia marcescens* colony and mutual capacitance images (D) of the same colony using the capacitance measured between pixel pairs of varied spacing. A reference electrode was not included for these measurements.

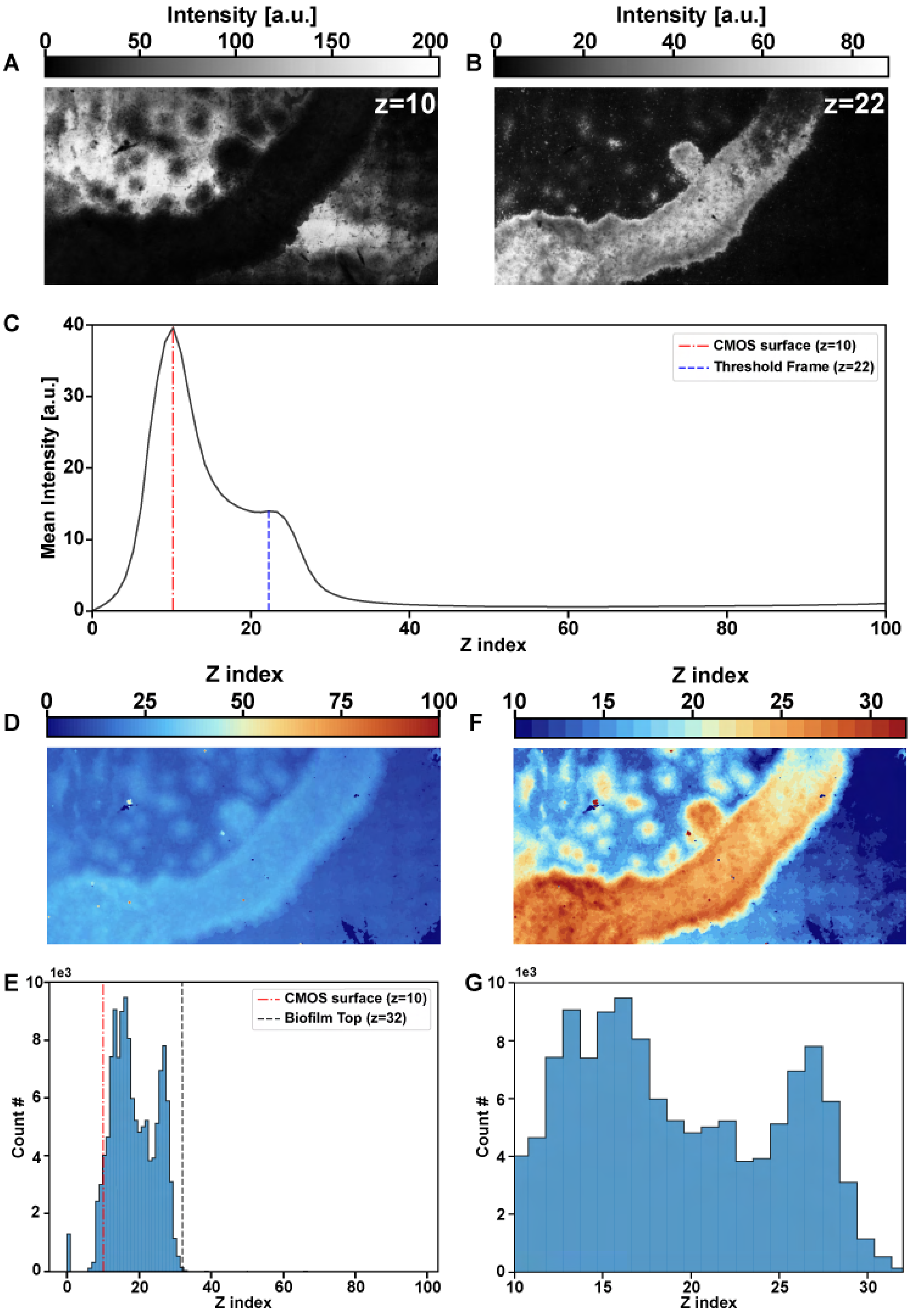

Supplementary Figure S5. Mapping biofilm thickness with 3D segmentation: (A) The plane of the CMOS array and (B) a frame for thresholding are determined from the mean intensity profile (C) across z indices. (D) A 2D heatmap of the maximum z positions where signal is present is produced using the threshold determined in (B). (E) The distribution of z value maxima reveals the highest point of the biofilm, as well as signal outliers detected both beneath the CMOS and above the sample in the media substrate. (F) The final heatmap of biofilm thickness and the corresponding distribution (G) after outliers are suppressed.

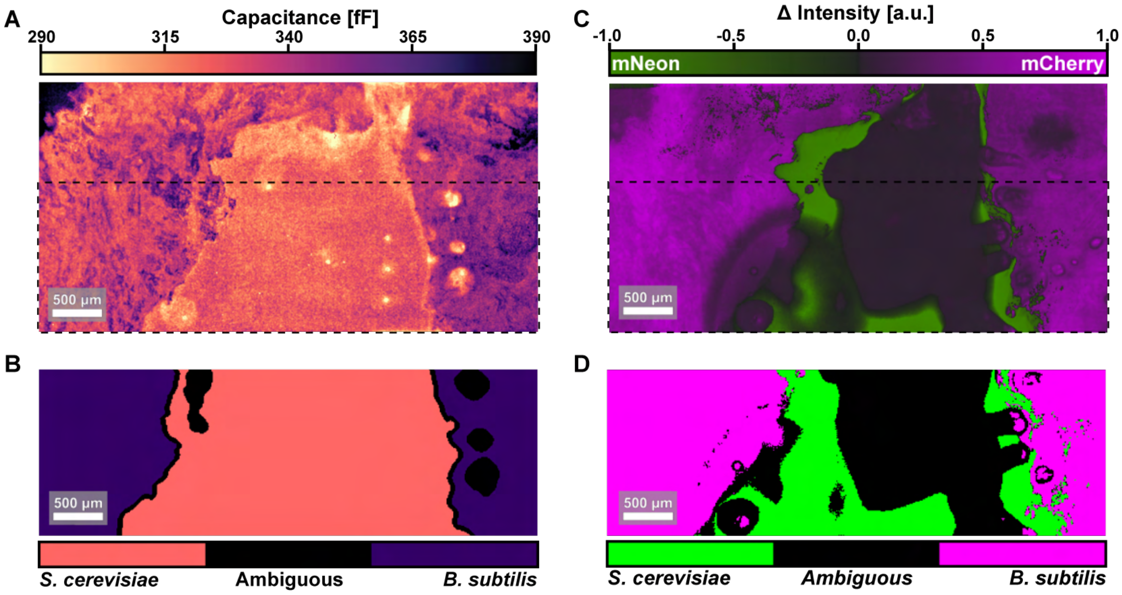

Supplementary Figure S6. Pattern Segmentation of multi-species ECI data: ECI data (A) is cropped to a region without media pockets, where we expect to measure only biomass from one of the two species.(B) A mask of the species locations is created by applying Otsu segmentation to the capacitance image of (A) with a Gaussian blur. Boundary regions where the two populations appeared ambiguously mixed were isolated using binary erosion, and three semicircular outlier regions were manually omitted (B, right). (C) A multichannel fluorescence image of the same region is used to identify the species of both populations (D). Notably, mNeon expression is weak in the center of the sample, yet the presence of biomass there is clear in ECI.

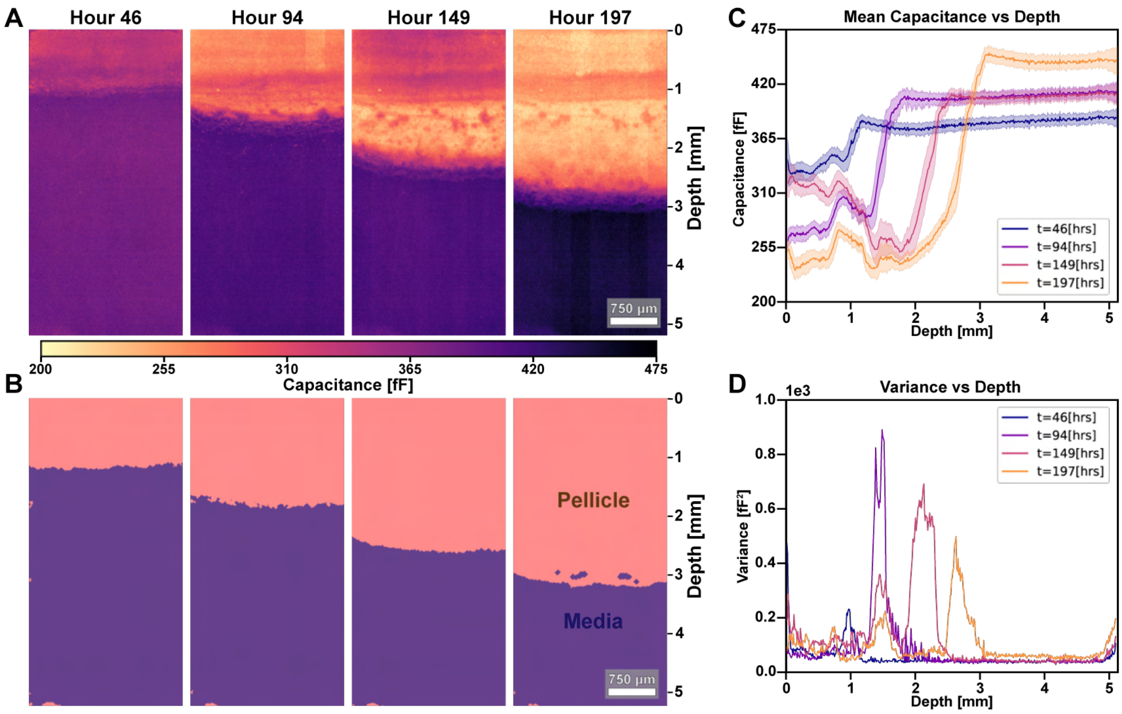

Supplementary Figure S7. Tracking pellicle formation in ECI time-lapse data: (A) ECI time-lapse data from Fig. 6 with the corresponding masks (B) identifying the expanding pellicle region. Traces of the capacitance mean with standard deviation (C) as a function of depth in the ECI images, and corresponding traces of variance (D).

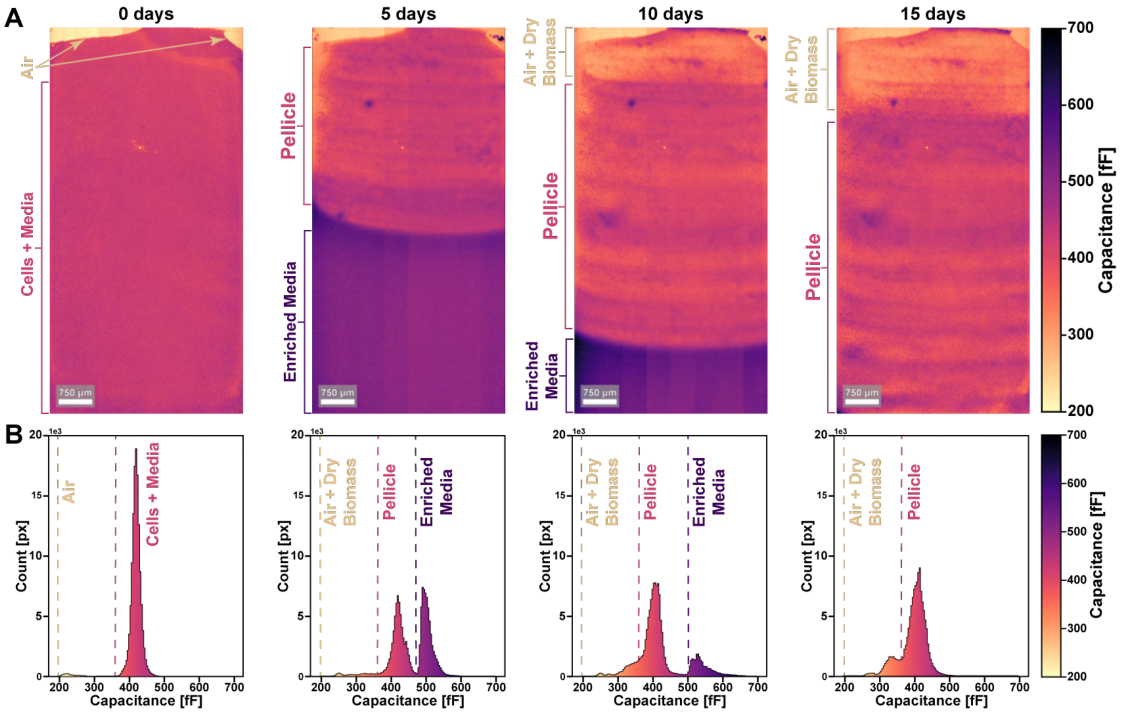

Supplementary Figure S8. Long term monitoring of pellicle development: (A) ECI data from a 16 day time-lapse captures the dynamics of pellicle formation at the liquid-air interface. (B) Histograms of the capacitance distribution corresponding to each time-point.

In Fig. S8A, we see the initially well mixed cell suspension (day 0) separates to form a pellicle at the air-liquid interface (day 5). Meanwhile, cell activity gradually causes electrolytic enrichment of the surrounding media, due to the continuous excretion of metabolites. As a result, culture media becomes increasingly distinguishable from pellicle biomass in the capacitance distribution (day 5 - 10), with media signal increasing in value relative to the signal of air and biomass (S8B), before eventually disappearing as pellicle growth exceeds the CMOS field of view (day 15). Evaporative losses slowly cause the liquid-air interface in the cuvette to recede downwards (day 10 - 15), which in turn causes biomass at the top of the pellicle to dry against the CMOS sensor. This dry biomass population can be seen across the histograms of Fig. S8B, emerging as a distinct peak between the values associated with the air and pellicle regions.

Supplementary Figure S9. Timelapse of Pellicle Development: A video of the pellicle development shown in Fig. 6 and S7 has been included with as supplementary material, with ECI data captured at 10 minute intervals.

LIST OF TABLES

S1 Species and Strain Information 1
S2 Sample information relevant to ECI/FL imaging 1
S3 Comparison of ECI and other methods. Sizes are determined from core imaging systems only, not including installation or shared peripheral equipment such as a measurement PC. Imaging times correspond to the time to capture an area equivalent to the CMOS field of view (2.56mm × 5.12mm) at the stated resolution. 2

LIST OF FIGURES

S1 **ECI acquisition hardware, and sample preparation:** (A) Data from the 131072 CMOS pixels is multiplexed into 8 readout channels, and streamed to a dedicated FPGA via a data-acquisition board. There it is decoded and transmitted via USB connection to a measurement PC, and saved in HDF5 file format. (B) The agarose sample of Fig 1D/E is seen mounted over the CMOS array, with an optional AgCl reference electrode inserted. (C) Views of the cuvette setup used in pellicle culturing, featuring the pellicle of Fig. S8 after several weeks of growth. (D) High level block diagram of system electronics, featuring the 8 readout channels. Multiplexed data from each channel undergoes Trans-impedance Amplification (TIA) followed by Programmable Gain Amplification (PGA), before signal is digitized with an 18-bit ADC on the data acquisition board, and finally decoded by the FPGA.3
S2 **Adjusting for channel readout artefacts:** (A) A capacitance image with an offset artefact from the 8 channel CMOS readout. (B) The capacitance correction to be applied to the image and (C) the corrected image. (D) Histogram of capacitance values before (E) and after the correction. (F) Demonstration of the effect of frame averaging on ECI (Fig. 1 data). Image quality is improved with averaging, but not substantially beyond 5x. 4
S3 **FFT suppression of CMOS features in optical images:** (A) The 2D FFT of a confocal image (left) with periodic features showing up at increments determined by the shape of the CMOS array (256,512). (B) A simple grid filter is applied in the frequency domain to suppress CMOS features.(C) The confocal image before (D) and after applying the FFT filter. 5
S4 **Self vs mutual capacitance modalities:** Our device is capable of multiple AC-coupled imaging modalities. (A) In a self-capacitance measurement, capacitance is measured between a target electrode and the rest of the array surface, while mutual capacitance measurements (B) can be made between specific electrode pairs in the array. We demonstrate these modalities with a self-capacitance image (C) of a *Serratia marcescens* colony and mutual capacitance images (D) of the same colony using the capacitance measured between pixel pairs of varied spacing. A reference electrode was not included for these measurements. 6
S5 **Mapping biofilm thickness with 3D segmentation:** (A) The plane of the CMOS array and (B) a frame for thresholding are determined from the mean intensity profile (C) across z indices. (D) A 2D heatmap of the maximum z positions where signal is present is produced using the threshold determined in (B). (E) The distribution of z value maxima reveals the highest point of the biofilm, as well as signal outliers detected both beneath the CMOS and above the sample in the media substrate. (F) The final heatmap of biofilm thickness and the corresponding distribution (G) after outliers are suppressed. 7
S6 **Pattern Segmentation of multi-species ECI data:** ECI data (A) is cropped to a region without media pockets, where we expect to measure only biomass from one of the two species.(B) A mask of the species locations is created by applying Otsu segmentation to the capacitance image of (A) with a Gaussian blur. Boundary regions where the two populations appeared ambiguously mixed were isolated using binary erosion, and three semicircular outlier regions were manually omitted (B, right). (C) A multichannel fluorescence image of the same region is used to identify the species of both populations (D). Notably, mNeon expression is weak in the center of the sample, yet the presence of biomass there is clear in ECI. 8
S7 **Tracking pellicle formation in ECI time-lapse data:** (A) ECI time-lapse data from Fig. 6 with the corresponding masks (B) identifying the expanding pellicle region. Traces of the capacitance mean with standard deviation (C) as a function of depth in the ECI images, and corresponding traces of variance (D). 9
S8 **Long term monitoring of pellicle development:** (A) ECI data from a 16 day time-lapse captures the dynamics of pellicle formation at the liquid-air interface. (B) Histograms of the capacitance distribution corresponding to each time-point. 10

S9 **Timelapse of Pellicle Development:** A video of the pellicle development shown in Fig. 6 and S7 has been included with as supplementary material, with ECI data captured at 10 minute intervals. 10
